# From Our Foreign Correspondents

**Published:** 1987-08

**Authors:** 


### From Our Foreign Correspondents

**Journey to Jabalpur**

The flight to Delhi was delightful. Thai Airlines do not guarantee to transport you to Heaven, but their air-hostesses do their best. Nothing, in my experience, fuels the fantasies of the middle-aged male better than the approach of a slim, curvaceous Siamese beauty in a hip-hugging sari, delicately proferring an orchid and a warm smile full of Eastern promise. I was still up in the clouds when we landed.

Delhi airport, by contrast, is not like Heaven. It is, I suppose, marginally better than Purgatory, but there's more delay. Several Jumbo-jets had landed in quick succession around midnight, and hundreds of travellers were crushed together in the humid Immigration Hall. We milled around hopefully, as one does at those mammoth cocktail parties where there's no food or drink and nobody wants to know you. There were a few ragged cleaners who sqatted on the floor and occasionally flapped a cloth at a grimy wall. There were also several groups of unshaven, cadaverous soldiers in long shabby greatcoats, who carried rifles and puffed furtively on their cigarettes, but these were the only signs of authority.

Eventually I found that part of the crowd had formed itself into a queue, which I joined. Fifteen minutes later, I reached a small table, behind which sat a bespectacled, white-bearded Sikh, clad in baggy shorts, khaki turban and a pullover with holes in it. He held out his hand and I gave him my passport. "Vair is your form? You must have a form". He sighed wearily and with an airy wave dismissed me back to the centre of the human maelstrom. After eddying around for another few minutes, I located another small table, behind which sat a plump lady in a sari. I asked politely if she knew where I could get an Immigration form. She inclined her head to one side and smiled sweetly. "Ve have run out, they vill be coming later". I began to grasp the essence of India.

The forms did of course arrive eventually, and I provided an acount, in triplicate, of my grandfather's profession, father's birthplace, mother's maiden name and similar details which should be invaluable to any future biographer. I was then able to rejoin the original queue, which was now even longer than before.

After passing this first hurdle, I was in due course confronted with the second, where another Immigration Officer checked up on the work of the first.

All this took some time, but I needn't have been impatient – we still had to wait an hour for the baggage.

The next job was to book my seat to Jabalpur, since I had been told (wrongly) that tickets for Indian domestic flights could not be purchased in the U.K. At 6.30 a.m. the booking-office opened, and I was pleased to find a queue of only 3 people. Astonishingly though, this queue never grew smaller. One small Hindu after another would come up to greet his long-lost friend at the front of the queue, or even recognize his neighbour the booking-clerk. After a long chat, a ticket would be purchased almost as an after-thought. None of these interlopers would admit to speaking any English, but at length a very dapper Sikh, whom I'd seen on the London plane, queue-jumped in the same way. I took out my grievances and aired them vigorously. He immediately distanced himself from his countrymen and became conspiratorial, "These people do that all the time. What can one do? That's India, my friend" and he patted me on the shoulder sympathetically.

I next queued to check-in, and on reaching the counter was sent away to queue for an excess baggage ticket, from whence I was in due course sent away to queue for the right change, and was then reprocessed through the queues again in the reverse order.

Next came the security-check, which to my surprise was very thorough. The passenger in front of me was a young Buddhist monk, resplendent in saffron robes, sandals and shaved head. He spoke neither English nor Hindi and appeared bewildered by the whole business. Each time he walked through the metal detector it triggered the alarm, and he was pulled back and invited, with much pantomime, to give up his metal objects. This was repeated about six times, and various knives, spoons, pill-boxes, prayer-wheels etc, were fished out one-by-one from the deepest recesses of his saffron underwear before the detector was satisfied. I had some difficulty explaining to him that his knives would be returned to him after the flight.

The plane to Jabalpur was small and cramped. Half of the passengers appeared to have T.B., the hostess had acne, and the barley-sugar sweets which she handed out did not inspire me with confidence that the airline was at the forefront of modern technology. But the view from the window made it all worthwhile. My father had been a soldier in India for most of the Second World War, and throughout my childhood I had received regular doses of his correspondence, Kipling, and cod-liver oil. All three had made a lasting impression. After all those years, the long-awaited sight of Mother India's varied landscape slowly unfurling itself below our wings was unforgettable.

Eventually we began our descent to Jabalpur, and my accommodation worry surfaced again. My invitation to lecture at the All-India Congress of Dermatology had been a bit vague. I had managed to establish the dates of the Conference, and (with more difficulty) the titles of my two lectures, but I had failed miserably in my attempt to get the address of either the Conference Hall or a suitable hotel. I just hoped someone would meet me at the airport.

We touched down in bright sunshine on what looked like a school playing-field, apart from the armed soldiers standing guard at intervals around the perimeter. A small coach bumped across the field, and took us to a small grass enclosure where the passengers were greeted enthusiastically by their friends. Slowly they all drifted off, and I was left standing, tired and forlorn, in a small field in the middle of India with no idea where I wanted to go next.

An elderly man dressed in a long overcoat, woolly scarf, gloves and balaclava helmet came over to me, slapping his arms. "Chilly this morning, isn't it?". The temperature was 60°F with no wind. He'd come from Madras, where anything less than 80°F is on the cool side. He was a dermatologist who was also going to the Conference, and he was most reassuring. "They vill always meet us. They know ven the plane comes, don't vorry". Another half-hour passed pleasantly and peacefully, then in the distance we saw the dust plume of a jeep driven towards us at high speed. Shouted commands volleyed round the scattered militia, and two Indian Army Officers scrambled out from a nearby hut. They were absolutely dazzling – shiny boots, knife-edge creases in their trousers, a cane tucked under the arm, ramrod spines, square shoulders, dazzling teeth and smart black tooth-brush moustaches. I saw why mother fell for a soldier. They paled into insignificance however compared with the magnificent creature who stepped from the jeep. A veritable blaze of Brigadier, with red shoulder tabs, gold trimmings, medals, ornate peaked cap, snappy swagger-stick, leather gloves, and of course a most vigorous grey moustache. After a good deal of shouting and stamping and saluting this eminence was led away by the acolytes, with a procession of the lower ranks dragging behind. It beat any Professorial ward round I've ever seen into a cocked-hat.

My medical friend and I, disappointed that the splendour had completely passed us by, continued to wait patiently. "Don't vorry, they vill always come to meet us, they know ven the plane comes".

Assorted cars came and went, but none for us. About 40 minutes later what looked like a small milk van arrived. Out stepped four very smartly dressed doctors – the President of the Conference, the Dean of the Medical School, the Professor of Medicine, and the Conference Organizer.

Things do happen in India – you just have to wait long enough.

J. L. Burton

**Medicine in the Mountains**

It has been known for Medical Societies to arrange to have their meetings in resorts where there seems little justification for the speciality to visit. No such reservations could apply to a recent meeting of the Anglo-French Medical Society. The theme of the meeting was Mountain Medicine and the venue was Chamonix at the foot of Mont Blanc. The society was formed in 1984 with the aim of bringing together members of the medical profession from France and Britain for the exchange of knowledge and experience. Previous meetings have been held in Bath, Vichy and Chester. The president is 'our man' Roger Celestin whose international reputation and mastery of the French language equip him ideally for this demanding role. Recently at a meeting at Frenchay he offered me the tempting bait of an insight into mountain medicine with some practical work on the slopes and of course I was unable to resist.

What I had not expected was the quality of the British contribution to the scientific programme, talking to the French in Chamonix about Mountain Medicine would be like carrying coals to Newcastle I thought. Not a bit of it. Of 13 papers delivered 6 were by British speakers. Two British papers were on mountain sickness from experience in the Himalayas and in the Andes, one on the psychological aspect which was found to play no part in the syndrome and the other on the effect of acetazolamide which enhanced acclimatisation and performance at high altitudes, and indeed was likened to taking several thousand feet off the top of Everest. There were also papers on experience with mountain rescue in Scotland, Wales and Lakeland. Of the French papers one from Chamonix on hypothermia stressed the importance of central rather than peripheral rewarming of victims and the use of a heart lung machine for the purpose. Other papers were on frostbite, snow blindness and other eye injuries. The latter especially apropos as we also saw a filmed reconstruction of the first ascent of Mont Blanc by Dr Michel Gabriel Paccard and Jacques Balmat made to commemorate the bicentenary of the event in 1786 when Paccard was afflicted with snow blindness. Other papers from the Chamonix team dealt with mountain injuries some of the most horrific being suffered by people who had fallen into crevasses. One victim had survived 7 days down a crevasse, but many, with multiple injuries, froze to death.

Our resourceful president had managed to get the British Council to sponsor a lecture by the President of the Royal College of Surgeons, Mr Ian Todd. The British Council exists to promote understanding abroad of Britain and its culture and this indeed was a very appropriate occasion. Mr Todd gave a most interesting lecture on the history of the Royal Colleges and the role that they have played in upholding high standards in the specialities.

The main disappointment was the weather. The most famous run at Chamonix is the Vallee Blanche. This is really a glacier which starts on the shoulder of Mont Blanc and runs down in a wide arc for about 14 miles to the valley below near Chamonix. It is an experience never to be forgotten, many of us were keen to do it and we were organised into parties of eight each with two guides. When the day came the clouds were low, visibility was nil and we could not go. My disappointment was modified later however when I learnt from one of the doctors at the hospital that so far this season they have had to rescue 64 people from falls into crevasses and that 6 of them, including two guides had died.

M. G. Wilson

